# Implementation of a Structured, Protocol-Based Hypertension Management Strategy in Romanian Primary Care: A Single-Arm Exploratory Study

**DOI:** 10.7759/cureus.109331

**Published:** 2026-05-21

**Authors:** Radu Tatar, Marius-Stefan Marusteri, Andreea Varga, Dragos-Gabriel Iancu, Diana-Andreea Moldovan, Raluca-Maria Tilinca, Ioan Tilea

**Affiliations:** 1 Department of Family Medicine, Faculty of Medicine, Doctoral School, George Emil Palade University of Medicine, Pharmacy, Science, and Technology of Târgu Mureș, Târgu Mureș, ROU; 2 Department of Medical Informatics and Biostatistics, Faculty of Medicine, George Emil Palade University of Medicine, Pharmacy, Science, and Technology of Târgu Mureș, Târgu Mureș, ROU; 3 Department of Family Medicine, Faculty of International Medical Studies, George Emil Palade University of Medicine, Pharmacy, Science, and Technology of Târgu Mureș, Târgu Mureș, ROU; 4 Department of Clinical Sciences - Internal Medicine VIII, Faculty of Medicine, Doctoral School, George Emil Palade University of Medicine, Pharmacy, Science, and Technology of Târgu Mureș, Târgu Mureș, ROU; 5 Department of Family Medicine, Faculty of International Medical Studies, Doctoral School, George Emil Palade University of Medicine, Pharmacy, Science, and Technology of Târgu Mureș, Târgu Mureș, ROU; 6 Doctoral School, County Clinical Emergency Hospital, George Emil Palade University of Medicine, Pharmacy, Science, and Technology of Târgu Mureș, Târgu Mureș, ROU; 7 Department of Clinical Sciences - Internal Medicine VIII, Faculty of Medicine, George Emil Palade University of Medicine, Pharmacy, Science, and Technology of Târgu Mureș, Târgu Mureș, ROU

**Keywords:** blood pressure control, cardiovascular risk, hypertension, implementation science, primary care, protocol-based management, romania, treatment optimization

## Abstract

Background: In Romania, blood pressure (BP) control remains suboptimal despite increasing treatment rates. Multicomponent interventions have been shown to improve BP control internationally but require adaptation for resource-constrained settings. This study assessed whether a structured intervention could improve BP control within the constraints of Romanian primary care.

Methods: This single-arm exploratory study was conducted in a primary care practice in Târgu Mureș, Romania, and consecutively enrolled 500 adults with uncontrolled hypertension (≥140/90 mmHg). A 12-month follow-up was completed in October 2024. The intervention comprised culturally adapted counseling (five to seven minutes for uncontrolled BP and two to three minutes for controlled BP), protocol-guided medication optimization, and specialist referral when clinically indicated. The primary outcome was achievement of target BP <140/90 mmHg at 12 months.

Results: Of the 500 enrolled participants with uncontrolled hypertension, 449 (89.8%) completed the 12-month follow-up. At study completion, 32.7% (95% confidence interval (CI) = 28.4-37.3) achieved target BP control, with mean reductions of 20.5 ± 9.2 mmHg in systolic BP and 10.3 ± 5.7 mmHg in diastolic BP. BP control rates differed substantially according to baseline hypertension severity, reaching 77.0% (95% CI = 69.6-84.3) among participants with Stage 1 hypertension compared with 15.5% (95% CI = 11.5-19.4) among those with Stage 2 hypertension (p < 0.001). In multivariable analysis, Stage 2 hypertension at baseline emerged as the strongest independent predictor of failure to achieve BP control (adjusted odds ratio (OR) = 0.05, 95% CI = 0.03-0.09; p < 0.001). Diabetes mellitus was associated with higher odds of BP control, although this association did not retain independent statistical significance after adjustment (adjusted OR = 1.64, 95% CI = 0.99-2.72; p = 0.055). Implementation of the structured intervention was associated with substantially higher rates of treatment optimization among participants who achieved BP control compared with those who remained uncontrolled (100% vs. 30.8%, p < 0.001).

Conclusions: A structured, protocol-based approach to hypertension management was feasible in routine Romanian primary care and achieved clinically meaningful improvements in BP control. Treatment optimization emerged as the key process associated with successful outcomes. These findings support the potential of scalable, low-resource interventions to improve hypertension control, but require confirmation in controlled, multicenter studies before broader implementation.

## Introduction

Hypertension remains the leading modifiable risk factor for cardiovascular disease globally and is a major contributor to premature mortality. In Romania, approximately 45% of adults are affected, and cardiovascular disease accounts for 57% of all deaths, representing one of the highest burdens within the European Union [1-3]. Despite the widespread availability of effective antihypertensive therapies, national data consistently show that only 30%-40% of treated patients achieve target blood pressure (BP) levels below 140/90 mmHg, falling short of the World Health Organization target of 50% control by 2025 [2,4,5]. This persistent gap between treatment and effective BP control represents a major contributor to preventable cardiovascular morbidity, mortality, and healthcare expenditure in Romania [6].

This discrepancy reflects a broader challenge in hypertension management, often described as a treatment-control gap, whereby increasing treatment rates do not translate into proportional improvements in BP control. The underlying causes are multifactorial and include therapeutic inertia, suboptimal treatment intensification, poor medication adherence, and structural limitations within healthcare systems. Addressing this gap requires not only effective pharmacological therapies but also strategies that improve their consistent implementation in routine clinical practice.

Primary care is the cornerstone of hypertension management in Romania, with family physicians responsible for the majority of diagnosed patients [7]. However, the delivery of guideline-recommended care is frequently constrained by organizational and structural limitations, including large patient panels, limited consultation time, and restricted access to specialist services [8]. These constraints reduce the feasibility of complex, resource-intensive interventions and necessitate pragmatic strategies that can be integrated into routine clinical workflows without requiring additional infrastructure. Similar challenges have been described across Central and Eastern European healthcare systems undergoing structural transition, supporting the broader relevance of findings generated in this context [9].

Evidence from meta-analyses and large-scale studies demonstrates that multicomponent interventions, typically combining behavioral counseling, pharmacological optimization, and structured follow-up, can improve BP control rates by 10-20 percentage points [10,11]. However, many of these interventions rely on additional personnel, digital infrastructure, or intensive monitoring strategies that are difficult to implement in routine primary care settings with limited resources. As a result, the key challenge is no longer to demonstrate efficacy under controlled conditions, but to identify which intervention components retain effectiveness when translated into real-world practice. In this context, implementation-focused approaches have gained increasing attention, emphasizing the translation of evidence-based interventions into routine care. Structured, protocol-based management strategies may reduce variability in clinical decision-making, support treatment optimization, and improve adherence to guideline recommendations, while minimizing additional workload for clinicians [12-14]. Despite this, there is a paucity of evidence evaluating simplified, time-efficient interventions delivered by a single physician within routine primary care, particularly in Central and Eastern European settings.

The present study aimed to evaluate the effectiveness of a structured, time-efficient, and culturally adapted multicomponent intervention implemented within routine Romanian primary care. The primary objective was to assess the proportion of previously uncontrolled hypertensive patients achieving BP control at 12 months. Secondary objectives included quantifying the magnitude of BP reduction, evaluating intervention implementation and utilization, identifying predictors of treatment success, and assessing safety outcomes.

## Materials and methods

Study design and setting

This single-arm exploratory study was conducted in a primary care practice in Târgu Mureș, Mureș County, Romania. Eligible patients were identified through systematic electronic health record review and invited to participate by the treating family physician, either by telephone or during scheduled routine consultations. Enrolled participants subsequently underwent a structured multicomponent hypertension management intervention with prospective 12-month follow-up until October 2024.

The study was specifically designed to evaluate the feasibility and real-world effectiveness of implementing a structured, protocol-based hypertension management strategy within routine primary care practice. Accordingly, the methodological approach prioritized external validity and practical applicability in everyday clinical settings rather than efficacy assessment under highly controlled experimental conditions [15].

The practice serves approximately 1,730 registered patients, with a predominantly urban population (76% urban, 24% rural). This distribution is more urban than that of Mureș County overall, where approximately 50% of the population resides in urban areas, and should be considered when interpreting the generalizability of the findings to rural populations.

Ethical approval was obtained from the Ethics Committee of the University of Medicine, Pharmacy, Science, and Technology of Târgu Mureș (approval no. 2505, October 13, 2023). All participants provided written informed consent prior to enrolment. The study was conducted in accordance with the Declaration of Helsinki and applicable Romanian data protection regulations.

Participants

Consecutive adult patients (≥18 years) with diagnosed essential hypertension and uncontrolled BP (≥140/90 mmHg) were enrolled during routine clinical visits. Daily electronic health record queries were used to identify hypertensive patients scheduled for consultation, ensuring systematic screening of all potentially eligible individuals.

The inclusion criteria were defined a priori and were as follows: 1) a documented diagnosis of essential hypertension for at least six months; 2) uncontrolled BP ≥140/90 mmHg confirmed by standardized measurement at the enrolment visit [16]; 3) regular attendance at the primary care practice, defined as at least two visits within the preceding 12 months; 4) a stable antihypertensive treatment regimen for a minimum of four weeks prior to enrolment, ensuring that elevated BP reflected persistent inadequate control rather than recent therapeutic modification; and 5) the ability to provide informed consent and to comply with follow-up procedures.

The exclusion criteria comprised the following: 1) suspected or confirmed secondary hypertension, including medication-induced causes identified through medical record review; 2) major cardiovascular events (myocardial infarction, stroke, or coronary revascularization) within six months prior to enrolment; 3) severe comorbid conditions limiting safe participation, including active malignancy, end-stage renal disease, or advanced hepatic failure; 4) cognitive impairment interfering with adherence to study procedures; 5) pregnancy or lactation; 6) anticipated relocation outside the practice catchment area within the follow-up period; 7) ongoing treatment with four or more antihypertensive drug classes at maximally tolerated doses; or 8) participation in another clinical study.

Intervention components

All participants received a structured, multicomponent intervention delivered by a single family physician investigator, ensuring consistent delivery and protocol fidelity. The intervention comprised three integrated components: behavioral counseling, pharmacological optimization, and structured specialist referral (see the Appendix).

Behavioral counseling was culturally adapted to the Romanian context and delivered in a time-efficient, structured format. Dietary recommendations focused on reducing sodium intake from commonly consumed sources, including processed meats, pickled foods, and high-fat cheeses, consistent with the sodium restriction targets and Mediterranean-style dietary principles recommended by the 2018 European Society of Cardiology (ESC)/European Society of Hypertension (ESH) hypertension guidelines, while physical activity guidance was structured to achieve at least 150 minutes per week of moderate-intensity aerobic exercise (≥30 minutes per session, five days per week) or at least 75 minutes per week of vigorous-intensity exercise over a minimum of three days, consistent with ESC/ESH lifestyle modification recommendations [17,18]. These targets were pursued through individually tailored activity plans, adapted to each patient's functional capacity, baseline activity level, and cultural context. Counseling intensity was tailored to BP status: patients with controlled BP (<140/90 mmHg) received brief reinforcement sessions (two to three minutes), whereas those with uncontrolled BP received structured sessions lasting five to seven minutes. Each session followed a predefined three-step algorithm: 1) identification of adherence and lifestyle barriers; 2) classification of the dominant barrier (medication-related or lifestyle-related); and 3) targeted counseling addressing the principal modifiable factor. Educational materials were provided in Romanian to support patient engagement and adherence.

Pharmacological management was guided by contemporary European hypertension recommendations, adapted to local drug availability and reimbursement policies [16,17]. Treatment optimization included systematic assessment of adherence barriers (e.g., cost, adverse effects, regimen complexity), stepwise dose titration based on tolerability, and addition or substitution of guideline-recommended drug classes, including angiotensin-converting enzyme inhibitors or angiotensin receptor blockers, calcium channel blockers, thiazide-like diuretics, and mineralocorticoid receptor antagonists.

Structured referral to specialist care (cardiology or hypertension services) was undertaken when clinically indicated, including in cases of resistant hypertension, suspected secondary hypertension, hypertensive urgency, or complex comorbidity [17]. The referral process was standardized through predefined criteria, facilitated appointment scheduling, and systematic follow-up to ensure completion and continuity of care.

Outcomes and measurements

The primary outcome was the proportion of participants achieving BP control at 12 months, defined as <140/90 mmHg according to the 2018 ESC/ESH and 2023 ESH guidelines for the management of arterial hypertension [16,17].

Secondary outcomes included the following: 1) absolute change in systolic and diastolic BP from baseline to 12 months; 2) mean arterial pressure, calculated as diastolic BP plus one-third of the pulse pressure: $\mathrm{DBP} + \frac{1}{3}\left(\mathrm{SBP} - \mathrm{DBP}\right)$, where DBP represents the diastolic blood pressure and SBP represents the systolic blood pressure; 3) time to first achievement of BP control; 4) intervention process indicators, including delivery of behavioral counseling, treatment optimization status, and completion of specialist referral; 5) medication-related outcomes, including dose adjustments, drug additions, and therapeutic substitutions; and 6) safety outcomes, including major adverse cardiovascular events (MACE), all-cause hospitalization, and treatment-related adverse effects.

BP measurements were obtained using a validated automated oscillometric device (Omron M3, Omron Healthcare, Kyoto, Japan) in accordance with standardized measurement protocols and current guideline recommendations [16]. All assessments were performed using an attended automated office BP measurement approach, with the treating physician present throughout the procedure. Participants were seated in a quiet environment and allowed a five-minute rest period before measurement initiation. Three consecutive BP readings were subsequently recorded at one-minute intervals, and the mean of the final two measurements was used for statistical analysis.

Safety outcomes were prospectively monitored throughout the 12-month follow-up period. MACE was defined as a composite of cardiovascular death, nonfatal myocardial infarction, nonfatal stroke, and hospitalization for heart failure.

Statistical analysis

As an exploratory, hypothesis-generating study, sample size was determined by operational feasibility rather than formal power calculation [18-21]. Constraints included the enrolment period, single-investigator design, and routine clinical workflow. The achieved sample of 500 patients was considered adequate to provide exploratory estimates with reasonable precision.

Continuous variables are presented as mean ± standard deviation (SD) for normally distributed data, or median (interquartile range (IQR)) for skewed distributions. Normality was assessed using the Shapiro-Wilk test and visual inspection of histograms. Categorical variables are presented as frequencies and percentages. Missing data were minimal (<2% for any variable).

The primary outcome, proportion achieving BP control at 12 months, is reported as a proportion with exact binomial 95% confidence intervals (CIs). Changes in BP were analyzed using paired t-tests or Wilcoxon signed-rank tests, depending on distribution [22]. Effect sizes for continuous outcomes were quantified using Cohen’s d (mean paired difference divided by the SD of the differences).

Univariable logistic regression analysis was performed to identify factors associated with achievement of target BP control at 12 months. The following baseline variables were evaluated: age, sex, residence (urban vs. rural), smoking status, hypertension duration, baseline hypertension stage (Stage 1 vs. Stage 2), baseline systolic BP (per 10 mmHg increase), diabetes mellitus, dyslipidemia, prior cardiovascular events, and number of antihypertensive agents at baseline.

Variables demonstrating an association at p < 0.10 in univariable analysis were considered eligible for inclusion in the initial multivariable model. Baseline hypertension stage and diabetes mellitus met this criterion and were therefore included in a multivariable analysis. Baseline systolic BP was not entered into the multivariable model due to collinearity with hypertension stage (Pearson r = 0.56); the categorical hypertension stage variable was retained because it provided greater clinical interpretability as a measure of baseline BP severity.

Treatment optimization and specialist consultation were intentionally excluded from multivariable modeling because these variables represented active components of the intervention and were considered potential mediators on the causal pathway between the structured management strategy and BP control. Including mediator variables in the regression model could have attenuated the estimated intervention-related associations and introduced overadjustment bias.

The final multivariable model was constructed using backward elimination with a retention threshold of p < 0.10. Results are reported as odds ratios (ORs) with 95% CIs. For subgroup analyses, age was additionally dichotomized at 65 years, consistent with ESC/ESH guideline definitions of older hypertensive patients for whom treatment targets may differ [16,17]. Group comparisons for categorical variables used chi-square tests or Fisher's exact tests when expected cell frequencies were below five. All analyses were performed using R statistical software (version 4.5.1; R Foundation for Statistical Computing, Vienna, Austria). No adjustment for multiple comparisons was applied, consistent with the exploratory nature of secondary analyses.

## Results

Participant flow

**Figure 1:**
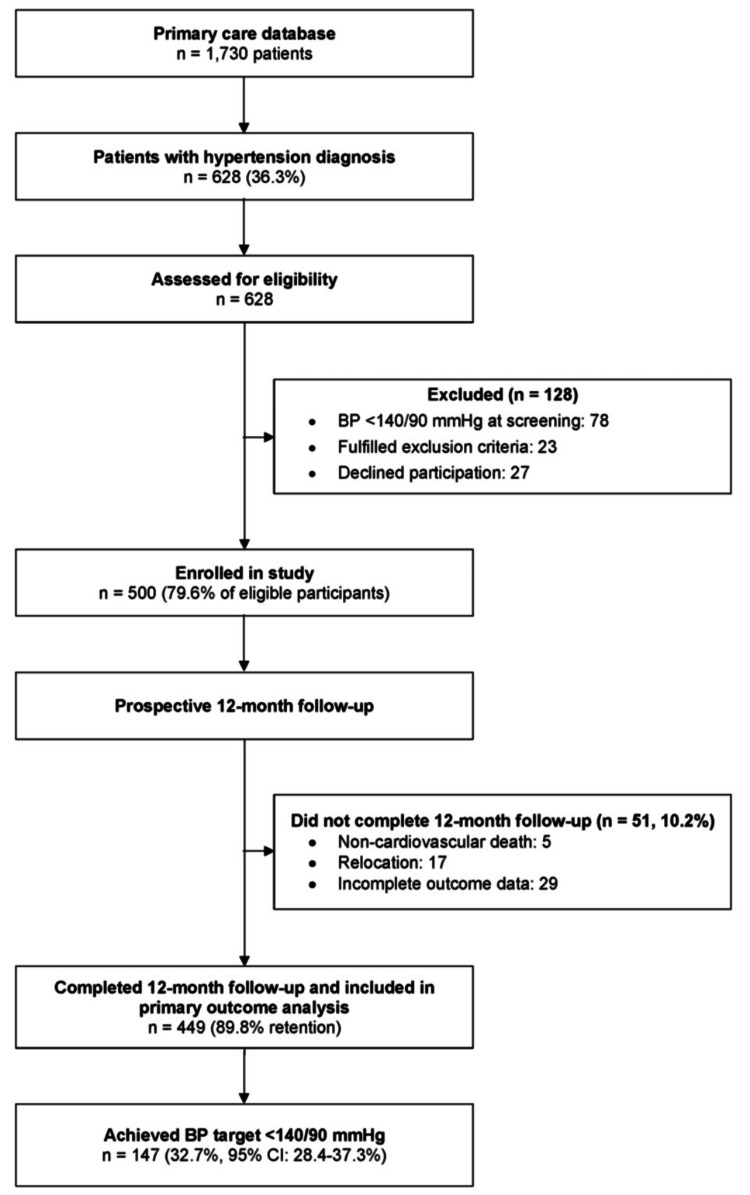
Patient flowchart BP, blood pressure

Of 1,730 active patients recorded in the electronic health record database, 628 (36.3%) had a documented diagnosis of hypertension and were screened for eligibility during routine clinical appointments (Figure 1). Among these, 128 patients (20.4%) were excluded: 78 (12.4%) had controlled BP at screening despite previous uncontrolled readings, 23 (3.7%) met one or more exclusion criteria, and 27 (4.3%) declined participation. Among those excluded due to eligibility criteria, the reasons included recent cardiovascular events (n = 8), suspected secondary hypertension (n = 6), severe comorbidities (n = 5), and cognitive impairment (n = 4). Among those declining to participate, the most frequently reported reasons were time constraints (n = 15), lack of interest (n = 8), or no stated reason (n = 4).

The remaining 500 patients were enrolled in the study, representing 79.6% of eligible individuals. During the 12-month follow-up period, 51 participants (10.2%) did not complete the study: five (1.0%) died from noncardiovascular causes, 17 (3.4%) relocated outside the practice catchment area, and 29 (5.8%) had incomplete outcome data due to missed final visits (n = 18) or unavailable specialist consultation records (n = 11). Consequently, the final analytic cohort comprised 449 participants, corresponding to an overall retention rate of 89.8% [23,24]. Baseline demographic and clinical characteristics did not differ significantly between completers and noncompleters, including age, sex, baseline BP severity, diabetes, and dyslipidemia status (all p > 0.15).

Baseline characteristics

Baseline characteristics of the analytic cohort are presented in Table 1. The median age was 63 years (IQR = 55-72), with 254 participants (56.6%) younger than 65 years. Women accounted for 264 participants (58.8%) and men for 185 (41.2%). Most participants resided in urban areas (n = 341, 75.9%), reflecting the location of the practice in Târgu Mureș. By study design, all 449 participants had uncontrolled hypertension at baseline. Median systolic BP was 161 mmHg (IQR = 151-171), and median diastolic BP was 96 mmHg (IQR = 90-104). Stage 2 hypertension (≥160/100 mmHg) was present in 323 participants (71.9%). Cardiometabolic comorbidities were frequent: diabetes mellitus was present in 158 participants (35.2%), dyslipidemia in 263 (58.6%), and prior cardiovascular events in 95 (21.2%). At baseline, participants were prescribed a median of two antihypertensive agents (IQR = 1-3).

**Table 1:** Baseline demographic and clinical characteristics of the analytic cohort (n = 449 patients) BMI, body mass index; BP, blood pressure; IQR, interquartile range; SD, standard deviation

Characteristics	Overall cohort (n = 449)
Demographic	
Age (years), median (IQR)	63 (55-72)
<65 years, n (%)	254 (56.6%)
Sex (female), n (%)	264 (58.8%)
Residence	
Urban residence, n (%)	341 (75.9%)
Hypertension history	
Duration (years), median (IQR)	9 (6-14)
Antihypertensive agents at baseline, median (IQR)	2 (1-3)
BP at baseline	
Systolic BP (mmHg), median (IQR)	161 (151-171)
Diastolic BP (mmHg), median (IQR)	96 (90-104)
Stage 2 hypertension, n (%)	323 (71.9%)
Cardiovascular risk factors and comorbidities	
Current smoker, n (%)	83 (18.5%)
BMI (kg/m²), mean ± SD	28.7 ± 3.6
BMI ≥30 kg/m², n (%)	170 (37.9%)
Diabetes mellitus, n (%)	158 (35.2%)
Dyslipidemia, n (%)	263 (58.6%)
Prior cardiovascular events, n (%)	95 (21.2%)

BP outcomes

At 12 months, BP control (<140/90 mmHg) was achieved in 147 participants (32.7%; 95% CI = 28.4-37.3). No participants met the control criteria at baseline per the study design. Mean systolic BP decreased from 161.8 ± 14.2 mmHg at baseline to 141.4 ± 15.1 mmHg at 12 months, corresponding to a mean reduction of 20.5 ± 9.2 mmHg (p < 0.001). Mean diastolic BP decreased from 98.3 ± 7.9 to 88.0 ± 9.7 mmHg, representing a mean reduction of 10.3 ± 5.7 mmHg (p < 0.001). Reductions in BP were observed progressively across follow-up timepoints (Table 2).

**Table 2:** Longitudinal changes in office blood pressure measurements over 12 months Values are presented in mmHg as mean ± SD. Δ values represent the mean change from baseline DBP, diastolic blood pressure; MAP, mean arterial pressure; SBP, systolic blood pressure; SD, standard deviation

Time point	SBP	DBP	MAP	ΔSBP	ΔDBP	Cohen's d (SBP)
Baseline	161.8 ± 14.2	98.3 ± 7.9	119.5 ± 7.1	-	-	-
1 month	154.9 ± 14.7	95.1 ± 8.7	115.0 ± 7.4	-7.0 ± 5.8	-3.2 ± 3.6	0.48
3 months	149.8 ± 15.3	92.3 ± 9.2	111.5 ± 7.9	-12.0 ± 7.3	-5.9 ± 4.3	0.81
6 months	146.6 ± 15.9	90.8 ± 9.7	109.4 ± 8.5	-15.3 ± 8.3	-7.5 ± 5.0	1.01
9 months	145.5 ± 15.9	90.3 ± 9.8	108.7 ± 8.5	-16.3 ± 8.3	-8.0 ± 5.1	1.08
12 months	141.4 ± 15.1	88.0 ± 9.7	105.8 ± 8.0	-20.5 ± 9.2	-10.3 ± 5.7	1.39

BP control according to baseline hypertension severity

BP control rates differed substantially according to baseline hypertension stage. Among 126 participants with Stage 1 hypertension, 97 (77.0%; 95% CI = 69.6-84.3) achieved BP control, compared with 50 of 323 participants (15.5%; 95% CI = 11.5-19.4) with Stage 2 hypertension (p < 0.001). Baseline BP levels were significantly lower among participants who achieved BP control than among those who remained uncontrolled (median = 153/92 vs. 166/101 mmHg; p < 0.001).

Among the 147 participants who achieved BP control, the median time to first controlled reading was six months (IQR = 3-12). Control was first documented at the one-month visit in 25 participants (17.0%), at the three- or six-month visit in 54 participants (36.7%), and at the 9- or 12-month visit in 68 participants (46.3%).

Intervention delivery and treatment optimization

All 449 participants received protocol-based behavioral counseling at each scheduled visit, resulting in a total of 2,694 counseling sessions. Mean session duration was 6.8 ± 1.2 minutes for visits with uncontrolled BP and 2.4 ± 0.6 minutes for visits with controlled BP.

Retrospective review of session documentation showed that 96% of sessions included all predefined core components: identification of adherence and lifestyle barriers, classification of the dominant barrier, targeted counseling, and follow-up planning.

Antihypertensive treatment adjustments were implemented in 380 participants (84.6%). Participants who remained uncontrolled at 12 months underwent treatment adjustments more frequently than those who achieved BP control (266/302, 88.1% vs. 114/147, 77.6%; p = 0.004). At 12 months, the median number of antihypertensive medications was higher among uncontrolled participants than among those who achieved BP control (3 (IQR = 2-3) vs. 2 (IQR = 1-3); p < 0.001), while both groups had a median of 2 medications (IQR = 1-3) at baseline. Across the full cohort, the distribution of antihypertensive medications shifted significantly between baseline and 12 months (median = 2 (IQR = 1-3) to 2 (IQR = 2-3); Wilcoxon signed-rank p < 0.001), reflecting increased treatment intensity among participants who remained uncontrolled.

End-of-study treatment optimization, defined according to ESC/ESH criteria, was achieved in 240 participants (53.5%) [16,17]. Optimization was present in all participants who achieved BP control (147/147) and in 93 of 302 participants (30.8%; 95% CI = 25.5-36.1) who remained uncontrolled (p < 0.001). The absolute difference in optimization rates between the groups was 69.2 percentage points.

A total of 134 participants (29.8%) met predefined criteria for specialist referral, of whom 116 (86.6%) completed the consultation. Referral coordination included facilitated appointment scheduling and systematic follow-up confirmation. Indications for referral were resistant hypertension (n = 78, 58.2%), complex comorbidity (n = 34, 25.4%), suspected secondary hypertension (n = 15, 11.2%), and hypertensive urgency (n = 7, 5.2%).

Predictors of BP control at 12 months

Univariable logistic regression identified several factors associated with BP control at 12 months (Table 3). Baseline hypertension severity was strongly associated with outcome: Stage 2 hypertension was present in 50 of 147 participants (34.0%) who achieved BP control compared with 273 of 302 participants (90.4%) who remained uncontrolled (OR = 0.05, 95% CI = 0.03-0.09; p < 0.001). Higher baseline systolic BP was also associated with lower odds of BP control (OR per 10 mmHg increase: 0.68, 95% CI = 0.59-0.78; p < 0.001).

**Table 3:** Univariable logistic regression analysis of predictors associated with blood pressure control at 12 months ^*^Median (IQR). Values are presented as numbers (percentages) unless otherwise specified. ORs are derived from univariable logistic regression models. The effect of baseline SBP is expressed per 10 mmHg increase BP, blood pressure; CI, confidence interval; HTN, hypertension; SBP, systolic blood pressure; OR, odds ratio; IQR, interquartile range

Variable	BP controlled (n = 147)	Uncontrolled BP (n = 302)	OR (95% CI)	p value
Age (per 10-year increase)	63 (57-71)^*^	62 (54-72)^*^	1.10 (0.92-1.30)	0.295
Sex (female)	82 (55.8%)	182 (60.3%)	0.83 (0.56-1.24)	0.365
Residence (urban)	115 (78.2%)	226 (74.8%)	1.21 (0.76-1.93)	0.430
HTN duration (per year)	10 (7-14)^*^	9 (5-13)^*^	1.01 (0.98-1.05)	0.413
Baseline SBP (per 10 mmHg increase)	153 (145-160)^*^	166 (155-175)^*^	0.68 (0.59-0.78)	<0.001
Stage 2 hypertension (SBP ≥160 and/or DBP ≥100 mmHg)	50 (34.0%)	273 (90.4%)	0.05 (0.03-0.09)	<0.001
Current smoker	21 (14.3%)	62 (20.5%)	0.65 (0.38-1.11)	0.112
Diabetes mellitus	62 (42.2%)	96 (31.8%)	1.57 (1.04-2.35)	0.031
Dyslipidemia	94 (63.9%)	169 (56.0%)	1.40 (0.93-2.10)	0.108
Prior cardiovascular events	36 (24.5%)	59 (19.5%)	1.34 (0.83-2.14)	0.229
Number of antihypertensive medications	2 (1-3)^*^	2 (1-3)^*^	0.95 (0.74-1.21)	0.665

With regard to clinical factors, diabetes was associated with higher odds of achieving BP control (OR = 1.57, 95% CI = 1.04-2.35; p = 0.031), while dyslipidemia and prior cardiovascular events were not significantly associated with outcome. Demographic characteristics, including age, sex, and residence, were likewise not significantly associated with BP control.

Among intervention-related variables, treatment optimization was present in all participants who achieved BP control compared with 93 of 302 participants (30.8%) who remained uncontrolled (p < 0.001). Completion of specialist consultation was also associated with higher odds of BP control (OR = 2.74, 95% CI = 1.87-4.44; p < 0.001).

Multivariable logistic regression with backward elimination identified Stage 2 hypertension at baseline as the only statistically significant independent predictor of failure to achieve BP control at 12 months (Table 4). Patients with Stage 2 hypertension had markedly lower odds of achieving BP control compared with those with Stage 1 hypertension (adjusted OR = 0.05, 95% CI = 0.03-0.09; p < 0.001). Diabetes mellitus showed a borderline trend toward higher odds of control in multivariable analysis (adjusted OR = 1.64, 95% CI = 0.99-2.72; p = 0.055), consistent in direction with the univariable estimate (OR = 1.57, 95% CI = 1.04-2.35; p = 0.031) but not independently statistically significant at p < 0.05 after adjustment for baseline BP stage. Treatment optimization and specialist consultation were excluded from the multivariable model as prespecified mediators in the causal pathway of the intervention.

**Table 4:** Multivariable logistic regression analysis of predictors associated with blood pressure control at 12 months ORs derived from multivariable logistic regression using backward elimination (retention threshold p < 0.10). Baseline SBP was excluded from the multivariable model due to collinearity with hypertension stage (r = 0.56). Model performance AUC-ROC = 0.799; McFadden R² = 0.277 CI, confidence interval; DBP, diastolic blood pressure; OR, odds ratio; SBP, systolic blood pressure; AUC, area under the curve; ROC, receiver operating characteristic

Variable	Adjusted OR	95% CI	p value
Stage 2 hypertension at baseline (SBP ≥160 and/or DBP ≥100 mmHg)	0.05	0.03-0.09	<0.001
Diabetes mellitus	1.64	0.99-2.72	0.055

Participants with diabetes achieved BP control more frequently than those without diabetes (62/158, 39.2% vs. 85/291, 29.2%; p = 0.031) and demonstrated a greater median reduction in systolic BP (22 mmHg (IQR = 16-28) vs. 19 mmHg (IQR = 14-26); p = 0.008).

Safety outcomes during 12-month follow-up

A total of 13 participants (2.9%; 95% CI = 1.6-4.9) experienced MACE during the 12-month follow-up. Events comprised nonfatal myocardial infarction (n = 5), stroke (n = 4), heart failure hospitalization (n = 3), and one cardiovascular death among study completers. No clustering of events was observed in relation to specific intervention components or treatment phases. This cardiovascular death was distinct from the five noncardiovascular deaths recorded among participants who did not complete follow-up.

All-cause hospitalization occurred in 36 participants (8.0%; 95% CI = 5.7-10.9), including hospitalizations due to cardiovascular causes (n = 13), infections (n = 9), elective procedures (n = 8), and other medical conditions (n = 6). Symptomatic hypotension, defined as systolic BP <90 mmHg with associated symptoms, occurred in seven participants (1.6%) and was managed by dose reduction. Tolerability-related medication adjustments were required in 10 participants (2.2%). No episodes of severe hypotension requiring hospitalization were recorded.

## Discussion

Study population and external validity

This single-arm exploratory study evaluated a structured, protocol-based hypertension management strategy implemented within routine Romanian primary care. Feasibility was high, with 79.6% of eligible patients enrolled and 89.8% completing 12-month follow-up. Attrition was primarily related to relocation and missed visits rather than clinical deterioration, and completers did not differ significantly from noncompleters in baseline characteristics, supporting the internal validity of the analytic cohort [23,24].

The study population reflects real-world primary care practice. Baseline characteristics, including age, hypertension severity, diabetes prevalence, dyslipidemia, and prior cardiovascular disease, were broadly consistent with Study for the Evaluation of Prevalence of Hypertension and Cardiovascular Risk in Adult Population in Romania III data for Romanian hypertensive populations, supporting the representativeness of the cohort [2]. The exclusion of 12.4% of identified patients due to controlled BP at chart review reflects real-world variability in BP status and further supports the pragmatic nature of the recruitment process. The predominance of women is consistent with known patterns of greater healthcare utilization among women [25]. However, the urban predominance reflects the practice's location in Târgu Mureș and may limit its generalizability to rural settings.

The high prevalence of Stage 2 hypertension at baseline (71.9%) suggests advanced disease at presentation and a substantial cardiovascular risk profile, consistent with delayed detection and suboptimal BP control in Romanian primary care [2,8].

BP reduction and clinical impact

Among patients with uncontrolled hypertension at baseline, 32.7% achieved BP control at 12 months. This proportion should be interpreted in the context of a cohort composed exclusively of previously uncontrolled individuals, which limits direct comparability with studies enrolling mixed populations that include patients already controlled at baseline [2].

The observed reductions in BP were clinically substantial, with mean decreases of 20.5 ± 9.2 mmHg in systolic BP and 10.3 ± 5.7 mmHg in diastolic BP. These reductions exceed those typically reported in primary care-based interventions and are of a magnitude associated with significant reductions in the risk of stroke, coronary events, and cardiovascular mortality in both epidemiological and interventional studies [10,11,26-29].

BP reduction followed a progressive trajectory without evidence of plateauing, suggesting a sustained rather than transient intervention effect. In a comparative context, a multifactorial hypertension management program such as Kaiser Permanente Northern California achieved substantially higher control rates but required considerable organizational infrastructure, whereas the Los Angeles barbershop trial reported higher absolute control rates through intensive pharmacist-led strategies delivered in nonclinical settings [30,31].

The achievement of a 32.7% control rate within a single-physician, low-resource primary care setting suggests that structured, guideline-aligned management may deliver clinically meaningful improvements without the need for extensive system-level redesign [10,11].

Impact of baseline hypertension severity and time to control

Baseline hypertension severity was a major determinant of outcome. Patients with Stage 1 hypertension achieved substantially higher control rates than those with Stage 2 hypertension (77.0% vs. 15.5%), indicating the importance of earlier identification and intervention [32].

From a population perspective, this finding aligns with Rose’s prevention paradigm, whereby shifting the distribution of risk through earlier intervention yields greater overall benefit than focusing solely on high-risk individuals [33]. Evidence from primary care settings similarly supports improved outcomes when treatment is initiated at lower baseline BP levels [17].

Time-to-control analysis further supports the need for sustained follow-up. Among responders, the median time to BP control was six months, and a substantial proportion required more than six months to reach target levels. This suggests that structured hypertension management should not be evaluated based solely on early response, but rather on sustained longitudinal engagement.

Treatment optimization, intervention delivery, and BP control

Treatment optimization, encompassing appropriate drug selection, dosing adequacy, rational combination therapy, and tolerability management, emerged as the strongest process-related correlate of BP control in this study. Optimization was present in all patients who achieved BP control compared with only 30.8% of those who remained uncontrolled, representing a 69.2 percentage-point difference.

Patients who remained uncontrolled underwent treatment adjustments more frequently than those who achieved BP control (88.1% vs. 77.6%, p = 0.004) and were prescribed a higher number of antihypertensive agents at study end (median 3 vs. 2, p < 0.001).

The modest increase in medication burden despite frequent adjustments supports a structured and targeted approach to treatment optimization rather than indiscriminate intensification. Despite this greater treatment intensity, BP control was not achieved, suggesting that treatment frequency and escalation alone were insufficient, and that the appropriateness and structure of pharmacological management may be more relevant than the number of medications prescribed. This observation aligns with evidence from team-based care models, nurse-managed protocols, and self-monitoring strategies, which demonstrate that structured, guideline-based management improves BP control in routine practice and that structured decision algorithms may help reduce the influence of cognitive shortcuts in clinical judgment [34-36]. However, given the observational design, these findings should be interpreted cautiously and do not allow mechanistic inference.

Beyond the adequacy and structuring of pharmacological therapy, several additional mechanisms may explain the persistent lack of BP control despite repeated treatment optimization. Clinical inertia, defined as the failure to intensify therapy despite unmet therapeutic targets and available clinical opportunity, remains a well-recognized obstacle in contemporary hypertension care [37]. The predefined treatment algorithm implemented in the present study was specifically intended to minimize this phenomenon, although its direct impact cannot be quantitatively assessed within the current study design.

Suboptimal adherence to progressively more complex antihypertensive regimens likely represented an additional determinant of persistent uncontrolled hypertension. Previous evidence consistently demonstrates declining adherence with increasing therapeutic burden, a pattern concordant with the higher median number of antihypertensive agents observed in participants who failed to achieve BP targets by study completion [14].

In addition, the presence of undiagnosed secondary hypertension among a subset of patients with apparent treatment resistance cannot be entirely excluded, despite systematic application of enrolment exclusion criteria and the use of structured referral pathways for clinically suspected cases. Finally, white-coat hypertension may have contributed to falsely elevated office-based BP measurements in certain participants, thereby leading to apparent noncontrol. This limitation is acknowledged within the study limitations section and could not be further explored in the absence of ambulatory or home BP monitoring data.

Patient-related barriers to adherence represented a clinically relevant component of the persistent nonresponse observed in this cohort, particularly in participants who underwent repeated treatment adjustments without achieving BP control. Identification of potential adherence barriers was systematically incorporated as a structured element of the predefined counseling algorithm and addressed during each clinical encounter. However, the frequency and specific types of barriers encountered were not collected as predefined study endpoints and are therefore discussed as qualitative clinical observations rather than formally measured outcomes [38].

From the treating physician’s perspective, the adherence-related challenges most consistently observed among nonresponders included low perceived necessity for continuous antihypertensive therapy in the absence of symptoms, concerns regarding long-term medication dependence, difficulty maintaining dietary sodium restriction within culturally embedded nutritional practices, and progressive reduction in treatment adherence with increasing pharmacological complexity. These barriers frequently coexisted in participants with apparent treatment resistance, further complicating the achievement of BP control despite structured pharmacological optimization. This observation is consistent with previous evidence demonstrating that adherence-related factors and clinical complexity interact in resistant hypertension and that effective management often requires targeted, individualized interventions that extend beyond protocol-based treatment intensification alone [14,38].

The behavioral component of the intervention was deliberately designed to be pragmatic and scalable. Brief, structured counseling sessions of five to seven minutes for uncontrolled BP, embedded within routine consultations, achieved high protocol fidelity (96%). The use of predefined decision algorithms may have reduced cognitive load for the treating physician, while cultural adaptation, including attention to locally prevalent dietary sodium sources, was intended to enhance patient relevance and engagement [39-43].

These findings suggest that structured intervention delivery, combining protocol-guided pharmacological optimization with brief, targeted behavioral counseling, can improve BP control within routine primary care without requiring additional personnel or complex infrastructure.

Predictors of BP control

Stage 2 hypertension at baseline was the dominant independent predictor of failure to achieve BP control, with an adjusted OR of 0.05 (95% CI = 0.03-0.09; p < 0.001). Only 15.5% of patients with Stage 2 hypertension achieved BP control at 12 months, compared with 77.0% of those with Stage 1, reflecting the substantially greater therapeutic burden associated with more severe baseline hypertension. This finding reflects the importance of earlier identification and intervention before progression to Stage 2 disease.

Diabetes mellitus was associated with higher odds of BP control in multivariable analysis (adjusted OR = 1.64, 95% CI = 0.99-2.72; p = 0.055). Although the direction and magnitude of the association remained consistent with the univariable estimate (OR = 1.57, 95% CI = 1.04-2.35; p = 0.031), the association did not retain independent statistical significance after adjustment for baseline BP stage. Participants with diabetes achieved BP control more frequently than those without diabetes (39.2% vs. 29.2%) and demonstrated greater reductions in systolic BP during the 12-month follow-up period.

The mechanisms underlying the observed association between diabetes and improved BP control cannot be established in the present study and should therefore be interpreted with caution. Potential contributing factors may include closer clinical surveillance, greater patient engagement with healthcare services, more frequent therapeutic reassessment, or differences in pharmacological management among patients with coexisting cardiometabolic disease.

These findings are consistent with previous evidence supporting the close interrelationship between diabetes management and BP control. Integrated treatment approaches targeting both conditions have been associated with additive cardiovascular risk reduction, particularly when supported by structured follow-up and protocol-driven care pathways [44-46].

Although exploratory and hypothesis-generating, the present observations raise the possibility that patients with diabetes may represent a subgroup particularly responsive to structured hypertension management interventions. Further prospective studies are warranted to confirm this association and to better elucidate the mechanisms involved.

Safety profile and clinical tolerability

During the 12-month follow-up, the incidence of MACE was 2.9% (13 participants; 95% CI = 1.6-4.9), a rate consistent with rates previously reported in treated hypertensive populations managed in primary care settings [18]. No temporal clustering of cardiovascular events was observed in relation to treatment modifications, specific intervention components, or phases of protocol implementation. Moreover, no cardiovascular events were considered attributable to the structured intervention itself or to protocol-guided BP management, suggesting the absence of an apparent excess cardiovascular risk associated with the study strategy.

All-cause hospitalization occurred in 36 participants (8.0%; 95% CI = 5.7-10.9), including admissions related to cardiovascular causes (n = 13), infections (n = 9), elective procedures (n = 8), and other medical conditions (n = 6). Importantly, no hospitalizations were attributed to hypotension or medication-related adverse effects requiring treatment discontinuation.

With respect to treatment tolerability, symptomatic hypotension, defined as systolic BP <90 mmHg accompanied by related symptoms, occurred in seven participants (1.6%) and was managed conservatively through antihypertensive dose reduction. Fewer than 3% of participants required medication adjustments because of tolerability-related adverse effects, and no episodes of severe hypotension requiring hospitalization were recorded during follow-up.

The intervention demonstrated an acceptable safety and tolerability profile throughout the study period, with no identifiable safety signals attributable to the structured management strategy. Nevertheless, the absence of a control group and the lack of independent event adjudication warrant cautious interpretation of these findings.

Economic and healthcare system implications

Although a formal cost-effectiveness analysis was not performed, the findings of this study have relevant economic implications. The intervention was delivered within routine primary care without additional personnel, specialized infrastructure, or digital systems, suggesting a low incremental cost of implementation. In settings such as Romania, where hypertension prevalence is high and healthcare resources are constrained, improvements in BP control may have substantial clinical and economic implications.

Given the magnitude of BP reduction observed, even modest improvements in BP control at the population level are expected to translate into meaningful reductions in cardiovascular events, healthcare utilization, and long-term costs. Evidence from large epidemiological and modeling studies indicates that reductions of this magnitude in systolic BP are associated with substantial decreases in the incidence of stroke, myocardial infarction, and heart failure, with corresponding economic benefits for healthcare systems [26-29].

In contrast to resource-intensive models, such as integrated health system programs or pharmacist-led interventions, the present approach relies on structured clinical decision-making and brief behavioral counseling embedded within existing consultations. This suggests that clinically meaningful gains may be achievable without substantial additional investment or system redesign.

From a health system perspective, scalable, protocol-based hypertension management strategies could represent a cost-efficient approach to addressing the high burden of uncontrolled hypertension in Romania and similar settings. Future studies should incorporate formal economic evaluations, including cost-effectiveness and budget impact analyses, to better quantify the potential value of such interventions [47-49].

Limitations

Several limitations of this study should be acknowledged. The single-arm design precludes causal inference, and the observed reductions in BP may have been influenced by regression to the mean, secular trends, increased adherence related to study participation (Hawthorne effect), or other unmeasured confounders.

The study was conducted in a single primary care practice and delivered by one physician, which may limit generalizability to other clinical settings, providers, and healthcare systems. Although baseline characteristics were broadly consistent with national data, the predominance of urban participants further limits extrapolation to rural populations [2].

The absence of an independent observer introduces the potential for performance and observer bias. The treating physician was also responsible for BP measurement and documentation of counseling fidelity, which may have influenced both intervention delivery and outcome assessment. Although a standardized measurement protocol and validated automated device were used, ambulatory or home BP monitoring was not incorporated, limiting the ability to account for white-coat or masked hypertension.

Treatment optimization and specialist referral were integral components of the intervention and were therefore appropriately excluded from multivariable modeling as potential mediators. While this approach is methodologically justified, it limits the ability to formally quantify their independent contribution to BP control. Additionally, treatment optimization was present in all 147 participants who achieved BP control, creating a complete separation scenario in logistic regression that would render its coefficient statistically nonestimable, providing a further analytical justification for its exclusion. The follow-up period of 12 months does not permit assessment of the long-term durability of BP control, persistence of behavioral change, or impact on hard cardiovascular outcomes.

Body mass index (BMI) was recorded as a baseline characteristic (mean = 28.7 ± 3.6 kg/m²; 37.9% of participants with BMI ≥30 kg/m²) but was not incorporated as a covariate in multivariable regression models or as a stratification variable in subgroup analyses. Although BMI was not significantly associated with BP control in univariable logistic regression (OR = 1.03 per unit BMI; p = 0.35), the inability to formally evaluate the contribution of adiposity to hypertension severity, treatment resistance, and variability in time to BP control represents a methodological limitation, particularly among participants requiring repeated treatment optimization without achieving target BP levels.

In addition, adherence-related barriers identified through the predefined counseling algorithm were not prospectively collected or analyzed as formal study endpoints. Consequently, the relative contribution of specific patient-related factors, including medication cost, treatment complexity, and low symptom-driven motivation, to persistent treatment nonresponse could not be quantitatively assessed.

Finally, adverse events were not adjudicated by an independent committee, and safety analyses were based on clinical documentation within routine practice. Although no excess safety signal was observed, these findings should be interpreted with appropriate caution.

Implications and future directions

The findings of this exploratory study support further evaluation of structured, protocol-based hypertension management across broader Romanian primary care settings. Multisite replication should be prioritized, particularly in rural practices, multiphysician practices, and healthcare environments with variable access to specialist services. Such studies are needed to assess reproducibility, implementation fidelity, and the contextual conditions under which the protocol remains feasible and clinically effective.

The more favorable response observed among patients with diabetes warrants dedicated investigation. Future studies should clarify whether this association reflects closer metabolic surveillance, greater patient engagement, more frequent therapeutic reassessment, or differences in pharmacological and cardiometabolic management. Understanding these mechanisms may inform integrated hypertension-diabetes care pathways and strengthen cardiovascular risk-reduction strategies in primary care.

From a health-system perspective, formal cost-effectiveness and budget impact analyses are required before policy-level translation. Future evaluations should consider direct medication costs, physician time, referral utilization, potential reductions in hospitalization, and the possible long-term prevention of major cardiovascular events within Romanian health technology assessment frameworks.

Future studies should also incorporate BMI, waist circumference, and validated adherence assessment instruments as standard baseline variables, allowing prospective evaluation of their contribution to treatment response and apparent treatment resistance. Scalability should be explored through task-sharing and digital support strategies, including nurse-assisted protocol delivery, home BP monitoring, structured follow-up reminders, and digital self-monitoring tools. These approaches should be evaluated within implementation science frameworks such as Reach, Effectiveness, Adoption, Implementation, Maintenance, with attention to reach, effectiveness, adoption, implementation fidelity, and long-term maintenance in routine primary care practice.

Finally, future adaptations of the protocol should consider alignment with the 2024 ESC Guidelines for elevated BP and hypertension, which update the 2018 ESC/ESH framework and emphasize simplified classification, diagnostic pathways, risk evaluation, and streamlined management strategies [50].

## Conclusions

In this single-arm exploratory study, implementation of a structured, protocol-based hypertension management strategy within routine Romanian primary care was feasible and associated with clinically meaningful improvements in BP control over 12 months. These findings suggest that guideline-aligned, low-resource interventions may improve hypertension outcomes in real-world primary care settings. However, given the exploratory design and absence of a control group, the results should be considered hypothesis-generating and require confirmation in controlled, multicenter studies, including formal evaluation of long-term outcomes and cost-effectiveness, before broader implementation can be recommended.

## Appendices

Structured counseling protocol - bilingual version (Romanian original/English translation)

PROTOCOL DE CONSILIERE (adaptat pentru practica clinică)

ID Pacient: _______   Data: _______   Medic: _______   Durata Sesiunii: _____ min

DECIZIA CLINICĂ INIȚIALĂ

Evaluarea rapidă a statusului TA (30 secunde) - Valorile TA actuale: ______/ ______ mmHg

Categoria pacientului:

☐  GRUP A: TA CONTROLATĂ (<140/90 mmHg) → Consiliere de întărire (2-3 minute)

☐  GRUP B: TA NECONTROLATĂ (≥140/90 mmHg) → Consiliere intensivă (5-7 minute)

GRUP A: CONSILIERE DE ÎNTĂRIRE PENTRU TA CONTROLATĂ (2-3 minute)

Recunoașterea succesului (30 secunde)

☐  Felicitări oferite: "Tensiunea dumneavoastră este excelent controlată!"

☐  Valorile comunicate: "___/___ mmHg este sub ținta de 140/90"

☐  Progresul recunoscut: "Eforturile dumneavoastră dau rezultate"

Întărirea comportamentelor pozitive (1-1.5 minute)

☐  Aderența la medicație confirmată: "Continuați să luați medicamentele exact ca până acum"

☐  Comportamentele sănătoase încurajate:

☐  "Mențineți dieta cu puțină sare"

☐  "Continuați activitatea fizică regulată"

☐  "Păstrați greutatea stabilă"

☐  "Vedeți că se poate - sunteți pe drumul cel bun!"

Monitorizarea simplă (30 secunde)

☐  Următoarea programare confirmată: Data: _______

☐  "Continuați exact ce faceți acum"

☐  "Sunați dacă TA crește peste 150/95"

GRUP B: CONSILIERE INTENSIVĂ PENTRU TA NECONTROLATĂ (5-7 minute)

FAZA 1: Problema și ținta (1 minut)

☐  "___/___ mmHg este peste ținta de 140/90"

☐  "Trebuie să scădem această tensiune pentru a vă proteja inima"

☐  "Obiectivul nostru este <140/90 mmHg"

☐  "De ce credeți că tensiunea nu s-a îmbunătățit?"

Răspunsul pacientului: _______________________________________________

FAZA 2: Identificarea rapidă a problemei principale (2-3 minute)

A. Verificarea medicației (45 secunde)

☐  Nu ia medicamentele zilnic → ACȚIUNE: Explicare importanță + strategii simple

☐  Ia medicamentele corect → ACȚIUNE: Medicația necesită ajustare

☐  Problemă de cost/efecte adverse → ACȚIUNE: Discutare alternative

☐  Decizia rapidă: Ajustare medicație DA / NU

Problema identificată: ________________________________________________

B. Factorul principal din categoria stil de viață (45 secunde)

☐  Sarea din mâncare (multe conserve, mezeluri)

☐  Lipsa exercițiilor fizice

☐  Greutatea în exces

☐  Fumatul

☐  Stresul

C. Consiliere țintită pe problema identificată (45 secunde)

☐  SAREA: "Eliminați sarea de pe masă și reduceți conservele/mezelurile la jumătate"

☐  EXERCIȚIILE: "Începeți cu 15 minute de mers pe jos zilnic, după-amiază"

☐  GREUTATEA: "Scădeți porțiile cu o treime și eliminați pâinea de la o masă pe zi"

☐  FUMATUL: "Sunați la linia de renunțare la fumat pentru ajutor"

☐  STRESUL: "Respirație profundă: 4 secunde inspirație, 7 secunde reținere, 8 secunde expirație"

FAZA 3: Planul simplu de acțiune (1-1.5 minute)

☐  O singură schimbare prioritară: "_________________________________"

☐  "Puteți să faceți această schimbare?"

☐  Bariera principală identificată: _________________________________

☐  Soluția simplă: _________________________________

☐  Controlul în: 2-4 săptămâni (Data: _______)

☐  "Dacă TA >180/110 sau simptome (dureri piept, amețeli severe) - sunați urgent"

Plan de acțiune în o propoziție: _________________________________________________

DOCUMENTARE SIMPLIFICATĂ

Pentru TA controlată (Grup A)

☐  Consiliere de întărire realizată; comportamente pozitive încurajate

☐  Pacientul motivat să continue: DA / NU

☐  Următoarea programare: _____ (interval normal de urmărire)

Pentru TA necontrolată (Grup B)

☐  Problema principală identificată: Medicație / Stil de viață / Ambele

☐  Schimbarea prioritară discutată: _________________________

☐  Ajustare medicație necesară: DA / NU

☐  Urmărire programată în: _____ săptămâni

☐  Pacientul înțelege planul: DA / NU

Durata totală a consilierii: _____ minute

GHID RAPID PENTRU DECIZIA MEDICAȚIEI

Dacă pacientul IA medicamentele corect:

☐  Prima opțiune: Creștere doză medicament actual

☐  A doua opțiune: Adăugare al 2-lea/3-lea medicament

☐  A treia opțiune: Referire la cardiolog

Dacă pacientul NU ia medicamentele regulat:

☐  Prima acțiune: Rezolvare bariere aderență

☐  A doua acțiune: Simplificare regim (combinații fixe)

☐  A treia acțiune: Programare vizită în 2 săptămâni

FRAZE CHEIE

Pentru TA controlată:

"Excelent! Tensiunea este perfect controlată!"

"Continuați exact ce faceți acum"

"Sunteți un exemplu pentru alți pacienți"

Pentru TA necontrolată:

"Să identificăm rapid ce împiedică scăderea tensiunii"

"Care e problema principală - medicamentele sau stilul de viață?"

"O singură schimbare mare poate face diferența"

"În 2-4 săptămâni vom vedea îmbunătățire"

Pentru motivație:

"Sănătatea inimii dumneavoastră depinde de acest lucru"

"Știu că puteți face această schimbare"

"Suntem o echipă în lupta cu tensiunea"

RESURSE RAPIDE

Alimente de evitat (Sodiu în cantitate mare):

Conserve (murături, fasole la conservă); mezeluri (salam, șuncă, carne în conservă); brânzeturi sărate (telemea, brânză de burduf); supă la plic; chipsuri, semințe sărate.

Exerciții simple recomandate:

Mers pe jos 15-30 de minute; urcatul scărilor; grădinărit; curățenie intensă; dans pe muzică.

Counseling protocol (adapted for clinical practice)

English Translation

Patient ID: _______   Date: _______   Physician: _______   Session Duration: _____ min

Initial Clinical Decision

Rapid assessment of BP status (30 seconds) - Current BP values: ______/ ______ mmHg

Patient category:

☐  GROUP A: CONTROLLED BP (<140/90 mmHg) → Reinforcement counselling (2-3 minutes)

☐  GROUP B: UNCONTROLLED BP (≥140/90 mmHg) → Intensive counselling (5-7 minutes)

GROUP A: REINFORCEMENT COUNSELLING FOR CONTROLLED BP (2-3 minutes)

Acknowledging Success (30 seconds)

☐  "Your blood pressure is excellently controlled!"

☐  "___/___ mmHg is below the target of 140/90"

☐  "Your efforts are paying off"

Reinforcing Positive Behaviours (1-1.5 minutes)

☐  "Continue taking your medications exactly as before"

☐  Healthy behaviours encouraged:

☐  "Maintain a low-salt diet"

☐  "Continue regular physical activity"

☐  "Keep your weight stable"

☐  "You can see it is working - you are on the right track!"

Simple Monitoring (30 seconds)

☐  Next appointment confirmed: Date: _______

☐  "Continue exactly what you are doing now"

☐  "Call if BP rises above 150/95"

GROUP B: INTENSIVE COUNSELLING FOR UNCONTROLLED BP (5-7 minutes)

PHASE 1: Problem and Target (1 minute)

☐  "___/___ mmHg is above the target of 140/90"

☐  "We need to lower this blood pressure to protect your heart"

☐  "Our goal is <140/90 mmHg"

☐  "Why do you think the blood pressure has not improved?"

Patient response: ___________________________________________________

PHASE 2: Rapid Identification of the Main Problem (2-3 minutes)

A. Medication Review (45 seconds)

☐  Not taking medications daily → ACTION: Explain importance + simple strategies

☐  Taking medications correctly → ACTION: Medication needs adjustment

☐  Cost/side effects issue → ACTION: Discuss alternatives

☐  Quick decision: Medication adjustment YES / NO

Problem identified: _________________________________________________

B. Main Lifestyle Factor (45 seconds)

☐  Salt in food (processed foods, cured meats)

☐  Lack of physical exercise

☐  Excess weight

☐  Smoking

☐  Stress

C. Targeted Counselling for the Identified Problem (45 seconds)

☐  SALT: "Remove the salt shaker from the table and halve your intake of processed and cured meats"

☐  EXERCISE: "Start with 15 minutes of walking daily, in the afternoon"

☐  WEIGHT: "Reduce portions by a third and eliminate bread from one meal per day"

☐  SMOKING: "Call the national smoking cessation helpline for help with quitting"

☐  STRESS: "Deep breathing: 4 seconds in, 7 seconds hold, 8 seconds out"

PHASE 3: Simple Action Plan (1-1.5 minutes)

☐  One priority change: "_________________________________"

☐  "Can you make this change?"

☐  Main barrier identified: _________________________________

☐  Simple solution: _________________________________

☐  Review in: 2-4 weeks (Date: _______)

☐  "If BP >180/110 or symptoms (chest pain, severe dizziness) - call urgently"

Action plan in one sentence: ___________________________________________________

SIMPLIFIED DOCUMENTATION

For controlled BP (Group A)

☐  Reinforcement counselling completed; positive behaviours encouraged

☐  Patient motivated to continue: YES / NO

☐  Next appointment: _____ (normal follow-up interval)

For uncontrolled BP (Group B)

☐  Main problem identified: Medication / Lifestyle / Both

☐  Priority change discussed: _________________________

☐  Medication adjustment required: YES / NO

☐  Follow-up scheduled in: _____ weeks

☐  Patient understands plan: YES / NO

Total counselling duration: _____ minutes

QUICK MEDICATION DECISION GUIDE

If the patient IS taking medications correctly:

☐  First option: Increase the dose of the current medication

☐  Second option: Add a 2nd/3rd medication

☐  Third option: Refer to cardiologist

If the patient IS NOT taking medications regularly:

☐  First action: Resolve adherence barriers

☐  Second action: Simplify regimen (fixed-dose combinations)

☐  Third action: Schedule next visit in 2 weeks

KEY PHRASES

For controlled BP:

"Excellent! Your blood pressure is perfectly controlled!"

"Continue exactly what you are doing now"

"You are an example for other patients"

For uncontrolled BP:

"Let us quickly identify what is preventing the blood pressure from coming down"

"What is the main problem - medications or lifestyle?"

"One major change can make all the difference"

"In 2-4 weeks, we will see improvement"

For motivation:

"Your heart health depends on this"

"I know you can make this change"

"We are a team in the fight against high blood pressure"

QUICK REFERENCE

Foods to Avoid (High Sodium):

Canned goods (pickles, canned beans); cured meats (salami, ham, tinned meat); salted cheeses (Romanian telemea cheese and burduf cheese - a traditional fermented sheep milk cheese); instant soups; crisps and salted snacks.

Simple Recommended Exercises:

Walking 15-30 minutes; climbing stairs; gardening; vigorous housework; dancing.
